# Video pulse rate variability analysis in stationary and motion conditions

**DOI:** 10.1186/s12938-018-0437-0

**Published:** 2018-01-29

**Authors:** Angel Melchor Rodríguez, J. Ramos-Castro

**Affiliations:** Department of Electronic Engineering, Group of Biomedical and Electronic Instrumentation, Universitat Politècnica de Catalunya, Jordi Girona, 1-3, 08034 Barcelona, Spain

**Keywords:** Heart rate variability (HRV), Pulse rate variability (PRV), Imaging photoplethysmography (iPPG), Sampling rate, Region of interest (ROI), Tracking algorithm, Stationary condition, Motion scenario

## Abstract

**Background:**

In the last few years, some studies have measured heart rate (HR) or heart rate variability (HRV) parameters using a video camera. This technique focuses on the measurement of the small changes in skin colour caused by blood perfusion. To date, most of these works have obtained HRV parameters in stationary conditions, and there are practically no studies that obtain these parameters in motion scenarios and by conducting an in-depth statistical analysis.

**Methods:**

In this study, a video pulse rate variability (PRV) analysis is conducted by measuring the pulse-to-pulse (PP) intervals in stationary and motion conditions. Firstly, given the importance of the sampling rate in a PRV analysis and the low frame rate of commercial cameras, we carried out an analysis of two models to evaluate their performance in the measurements. We propose a selective tracking method using the Viola–Jones and KLT algorithms, with the aim of carrying out a robust video PRV analysis in stationary and motion conditions. Data and results of the proposed method are contrasted with those reported in the state of the art.

**Results:**

The webcam achieved better results in the performance analysis of video cameras. In stationary conditions, high correlation values were obtained in PRV parameters with results above 0.9. The PP time series achieved an RMSE (mean ± standard deviation) of 19.45 ± 5.52 ms (1.70 ± 0.75 bpm). In the motion analysis, most of the PRV parameters also achieved good correlation results, but with lower values as regards stationary conditions. The PP time series presented an RMSE of 21.56 ± 6.41 ms (1.79 ± 0.63 bpm).

**Conclusions:**

The statistical analysis showed good agreement between the reference system and the proposed method. In stationary conditions, the results of PRV parameters were improved by our method in comparison with data reported in related works. An overall comparative analysis of PRV parameters in motion conditions was more limited due to the lack of studies or studies containing insufficient data analysis. Based on the results, the proposed method could provide a low-cost, contactless and reliable alternative for measuring HR or PRV parameters in non-clinical environments.

## Background

Heart rate variability (HRV) is a physiological parameter that has gained importance due to its relations with the autonomic nervous system (ANS) and cardiovascular disorders. HRV is defined as the oscillation in the time interval between consecutive heartbeats (RR) [1]. It is considered to be an excellent indicator of health status due to its advantages of being objective, reliable, and easy to obtain [2]. In general, a high HRV is related to a good health status, wellness, and optimal adaptation to physical activity performance [3]. Conversely, a low HRV is related to cardiovascular disorders, poor fitness, and a non-adaptation to physical and psychological stress [4, 5].

In the last few years, some studies have measured heart rate (HR) or HRV parameters [1] using a video camera [6–9]. This technique is known as video-imaging photoplethysmography (iPPG) and focuses on the measurement of the small changes in skin colour caused by blood perfusion. Diverse factors may influence this method, such as anatomical and physiological characteristics of the person involved, the lighting conditions or the sensor properties of the cameras.

Although this research area has attracted great interest for being a low-cost method that is contactless and easy to implement, the number of studies has so far been very limited. Most of the previous works have obtained HRV parameters in stationary conditions, and there are practically no studies on motion scenarios that perform an in-depth statistical analysis of the data. These works have carried out different measurement set-ups, image and signal processing, and the statistical analysis of data. Moreover, some authors have investigated the possibility of measuring pulse rate variability (PRV) as a surrogate of HRV showing close agreement [10, 11]. Thus, a PRV analysis constitutes a good, reliable, and more comfortable alternative for measuring HRV.

One of the first works that used this technique is the study presented by Takano and Ohta [12]. In this work, the video recordings consisted of analog images from a charge-coupled device (CCD) camera, which were obtained in a sitting posture. The iPPG signal was obtained by measuring the changes in the average brightness within a region of interest (ROI) on the subject’s face. HR measurements were obtained by carrying out a frequency-domain analysis. Subsequently, Poh et al. [13] applied the method of blind source separation by independent component analysis (ICA) to obtain the iPPG signals. This study was conducted in stationary and motion conditions. The algorithm based on work by Viola and Jones [14] and Lienhart and Maydt [15] was used to detect the ROI. HR was measured in 30-s moving windows with 1-s of overlap by applying the Fast Fourier Transform (FFT). Along the same lines, Monkaresi et al. [16] proposed an algorithm for measuring HR in sitting still and naturalistic human–computer interaction (HCI) scenarios. The aim of the study was to improve the HR estimation by employing the Poh et al. method [13] in combination with machine learning techniques: linear regression and k-nearest neighbor (kNN). HR measurements were also extracted from 30-s moving windows with 1-s of overlap by a power spectral analysis.

Other works that also used the video imaging technique have measured the interbeat intervals (IBIs) or HRV parameters [17–26]. Poh et al. [17] presented a multiparameter physiological measurement work in which HR and HRV parameters were obtained by using the ICA method. Unlike in their previous work, HR measurements were estimated by calculating the mean of the IBIs. The frequency-domain components were estimated by a power spectral density (PSD) using the Lomb periodogram. Alternatively, Sun et al. [18] conducted a PRV analysis focused on the palm of the subject’s hand. Since the measurements obtained from this body part are also affected by the motion, the study was performed in stationary conditions by using a cushion placed under the hand to minimize artifacts. However, a procedure of reduced frames by pixel averaging was carried out to attenuate small motion artifacts. The iPPG signals were obtained from each averaged position across the sequence of reduced frames. Subsequently, a technique based on wavelet transforms was employed to detect the pulse-to-pulse (PP) intervals. Time and frequency domain parameters were obtained as part of the PRV analysis.

McDuff et al. [19, 20] have proposed the use of a five band digital camera with the aim of evaluating alternate combinations of frequency bands that yield better results in the measurement of physiological parameters. Correlations for all combinations of the colour channels were calculated in [19]. The measurements of both works were obtained at rest and under stress conditions. In another study, Moreno et al. [21] conducted a HRV analysis in supine and sitting postures with controlled illumination, synchronized breathing and eyes closed. This work presented a cross-correlation analysis with the aim of finding the face areas on averaged frames that could provide more information on HR. Some of the most common time and frequency domain parameters of HRV were reported in the study. Alghoul et al. [22] presented a comparison between two approaches to measure HRV parameters from the face in stationary conditions. These approaches are based on the ICA and Eulerian Video Magnification (EVM) methods, respectively.

In addition to stationary conditions, some authors also have conducted the acquisition of the IBIs in motion scenarios. Bousefsaf et al. [23] proposed a motion-tolerant method to measure the instantaneous HR. This method employs a skin detection filter and the u* component of the CIE L*u*v* colour space to improve its robustness in presence of motion or illumination changes. A wavelet-based filtering is applied in order to remove noise components from the raw iPPG signals. The recordings were obtained at rest and with predefined head movements. The study of Kumar et al. [24] proposed a method of combining skin-colour change signals from different regions of the face. This method used a weighted average to improve the signal-to-noise ratio (SNR), and the weights depended on the blood perfusion and the incident light intensity in the region. PP interval estimations were carried out in stationary, reading, watching video, and talking scenarios. HRV parameters were not obtained in the study.

Antink et al. [25] performed a beat-to-beat estimation by using different signals and their fusion. The assessed signals were obtained from the changes of the skin colour and the head motion, both by video, and from a ballistocardiographic mat sensor, which were fused using a Bayesian approach. Evaluations of each signal and their fusion are presented in the paper. The trials performed in this study were: sitting still, reading without motion, and reading without further instructions. Huang and Dung [26] proposed the application of the chrominance-based remote PPG (C-rPPG) method, followed by a continuous wavelet transform (CWT)-based denoising technique. A data acquisition procedure was carried out before employing C-rPPG and CWT. The procedure included face and skin colour detection, the computing of the averaged RGB values within the ROI and the upsampling of the signals. The face tracking was performed by means of nose detection for purposes of robustness. HRV parameters were obtained in static postures and occasional/frequent motion. A summary of the measurement set-up, signal domain analysis, and results of the previous cited works is presented in Table 1.

**Table 1:** Summary of the measurement set-up, signal domain analysis and results of reference works

References	Activity condition/method	Subj. N	Recording time	Resolution (fps)	Signal analysis	Overall results
[13]	1 = sitting still 2 = natural movements (no large or rapid movements)	12	1 min	640 × 480 (15 fps)	FD	
[16]	1 = sitting still 2 = natural human-computer interaction	10	(1) 1 min (2) 20 min	640 × 480 (30 fps)	FD	
[17]	Sitting still in front of a laptop	12	1 min	640 × 480 (15 fps)	TD, FD^†^	
[18]	Resting conditions (palm of the subject’s hand)	10	4 min	384 × 256 (200 fps)	TD, FD^†^	
[19]	1 = sitting at rest 2 = sitting under stress	(1) 9 (2) 10	2 min	960 × 720 (30 fps)	TD, FD^†^	
[20]	1 = sitting at rest 2 = sitting under stress	11	2 min	960 × 720 (30 fps)	TD	
[21]	1 = supine position 2 = sitting position (controlled illumination, synchronized breathing and eyes closed)	(1) 12 (2) 8	5 min	640 × 480 (30 fps)	TD, FD^†^	
[22]	Sitting still in front of a camera (1 = ICA-based method 2 = EVM-based method)	12	2 min	720 × 480 (30 fps)	TD, FD^†^	
[23]	1 = sitting still and calm 2 = sitting with pre-defined head movements	12	35 s	320 × 240 (30 fps)	TD	
[24]	1 = stationary 2 = reading 3 = watching video 4 = talking	5	80 s	1280 × 1024 (30 fps)	TD	
[25]	1 = sitting still 2 = reading without motion 3 = reading without further instructions	4	2 min	800 × 600 (30 fps)	TD	
[26]	1 = static 2 = static with makeup 3 = occasional motion 4 = frequent motion	(1) 4 (2) 3 (3) 3 (4) 2	1 min	640 × 480 (30 fps)	TD	

*FD* frequency-domain, *TD* time-domain (NN or PP time series), *r* Pearson correlation coefficient, *ρ*_*c*_ concordance correlation coefficient, *n.u.* normalized units

* p < 0.001; ^†^only for the acquisition of frequency-domain parameters of HRV; PRV: pulse rate variability (time series between consecutive pulse beats). The MAE results from [25, 26] are calculated in this work to obtain a mean value of the individual results (note: the recordings of the occasional and frequent motion categories [26] were performed by the same subject). For comparison purposes, only were included the parameters that were calculated in our study

The state of the art shows that most of the works have obtained HRV/PRV parameters in stationary conditions, and there are practically no studies that obtain these parameters in motion scenarios and by conducting an in-depth statistical analysis of the data. Motion is a relevant factor that must be considered because it could significantly affect the measurements by video imaging. Therefore, this present work proposes a selective tracking method using the Viola–Jones and KLT algorithms, with the aim of carrying out a robust video PRV analysis in stationary and motion conditions. This face tracking approach is more efficient and less computationally expensive than the Viola–Jones algorithm alone, as applied in some reference works. These advantages are particularly attractive for a potential real-time implementation in mobile platforms. Furthermore, given the importance of the sampling rate in a PRV analysis and the low temporal resolution of commercial cameras, we carried out an analysis of two models to evaluate their performance in the measurements. The results are decisive for the choice of an appropriate camera for conducting a more reliable PRV analysis by video imaging. Several statistical parameters and plots were calculated to evaluate the results obtained by the proposed method, as well as contrasting them with data reported in related works. The larger sample size and the in-depth statistical analysis of our study provided greater reliability of the data. The results of HR and PRV parameters were improved by our method in comparison with data reported in the state of the art.

## Methods

### Data acquisition

**Fig. 1:**
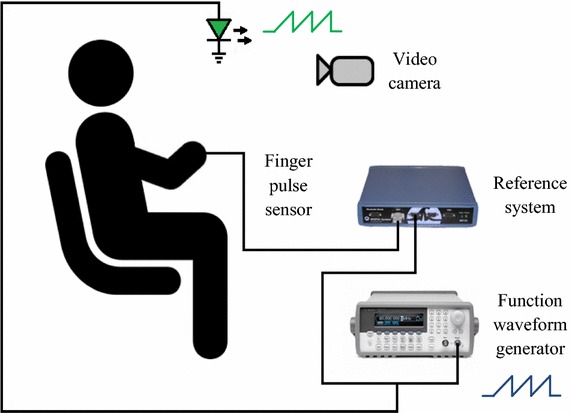
Data acquisition set-up of the study

The data acquisition was performed on a simultaneous recording of a video camera and a reference system in a sitting position. A representation of this configuration is illustrated in Fig. 1. A total of 15 healthy volunteers, 12 men and 3 women, participated in the measurements with the provision of informed consent. The data of the subjects corresponding to age, weight, height and body mass index (BMI) were (mean [min, max]): age (years): 26 [23, 35]; weight (kg): 69.7 [45, 86]; height (m): 1.72 [1.56, 1.87]; BMI: 23.4 [18.5, 27.4]. All subjects who participated in the study have fair skin with very slight tone variations among them. The study was conducted according to the principles defined in the Declaration of Helsinki. Two videos of about 1 min were obtained in all subjects at a distance of 0.3 m between the camera and the face. 50 s of the recordings were considered in order to analyse the same recorded length in all subjects. The settings of the video cameras are outlined in “First study: performance analysis of video cameras” section.

Firstly, we recorded a video asking the participants to remain still throughout the acquisition. Since the application of a tracking algorithm and the influence of motion are under evaluation in this study, it is necessary to conduct an analysis in stationary conditions for comparison. Secondly, we asked the participants to perform lateral and forward/backward movements, always looking toward the camera and avoiding fast gestures. Recordings were carried out indoors with sunlight as the only source of illumination [27] in order to avoid any artificial source that might contribute with high-frequency artifacts.

A reference system (MP36 BIOPAC Systems Inc.) was used to record the reference pulse signal. Two input channels were used for the acquisitions: the first one was used to record the reference signal with a finger pulse sensor at a sampling frequency of 1 kHz, and the second to record a sawtooth signal of 1 Hz obtained from a function waveform generator (Agilent 33220A) to facilitate the synchronization of the pulse signals (refer to “Synchronisation of the pulse signals” section).

### Studies

#### First study: performance analysis of video cameras

The first study consisted of a comparative analysis of two commercial cameras with different features in order to evaluate their performance in the measurements. In this part of the study, 5 subjects (4 men and 1 woman) were analysed in stationary and motion conditions. One of the cameras (GoPro HERO3 silver edition) is a versatile model that has been widely used lately in different scenarios and has the capability of diverse video resolutions. Depending on the resolution, it is possible to record videos at different frames per second (fps). We chose a resolution of 1280 × 720 pixels to record at 60 fps, a higher number of fps than most of the commercial video devices are able to record. The aim of recording at a higher frame rate was to assess its influence on the measurements.

The second camera (Logitech HD Pro Webcam C910) also has the capability of different video resolutions, but with a lower number of fps. In this model, we chose a resolution of 1920 × 1080 pixels at 15 fps, which is the maximum frame rate achievable at this video resolution. We chose this camera because it enables us to adjust diverse parameters manually. The focus, gain and exposure time were fixed in order to obtain an adequate focus and illumination of the face. The fixing of these parameters also avoids automatic adjustments caused by the movements of the subject or to ambient light changes. This manual configuration was not possible in the GoPro model.

#### Second study: PRV analysis

Once a camera was chosen on the grounds of providing a better performance, we carried out a PRV analysis in stationary and motion conditions. A total of 15 subjects (12 men and 3 women) were analysed in the study. Recordings acquired in the first study were included in the analysis. The results were contrasted with the data reported in previous works in similar conditions.

### ROIs detection and tracking

We used the Viola and Jones [14] and KLT [28, 29] algorithms to perform ROIs detection and tracking. The algorithms enable regions of the face to be detected and the tracking of feature points along the video sequence, respectively. Some problems such as multiple, incorrect or misdetections of face or eyes arose with the use of the Viola and Jones algorithm. Some conditions were then established on the basis of the four-element vector specified in pixels, which define the top-left coordinate (*x, y*), width (*w*), and height (*h*) of the ROI. The *w* and *h* of the ROI of the face (*ROIf*) and eyes (*ROIe*) were verified within the following pixel values:

$$\begin{aligned} 450 & > ROIf_{w} > 800,\quad 450 > ROIf_{h} > 800 \\ 250 & > ROIe_{w} > 550,\quad 50 > ROIe_{h} > 150 \\ \end{aligned}$$

If the *w* or *h* value failed to meet the criterion, the ROI was rejected. In this case, a new detection was carried out in the following frame. If both ROIs were within the established values, the four-element vectors [*x*, *y*, *w*, *h*] were modified according to the following percentages:

$$\begin{aligned} ROIf & = \left[ {ROIf_{x} + 0.2\;ROIf_{w} , 1.1\;ROIf_{y} , 0.6\;ROIf_{w} , 0.6\;ROIf_{h} } \right] \\ ROIe & = \left[ {ROIe_{x} \;{-}\;0.1\;ROIe_{w} , 0.85\;ROIe_{y} , 1.2\;ROIe_{w} , 1.8 \, ROIe_{h} } \right] \\ \end{aligned}$$

The coordinates were modified with the aim of analysing only the forehead and cheek regions. In particular, these areas have a major blood perfusion in the face, and thus we were able to obtain more information on the pulse rate. Moreno et al. [21] showed that the forehead and cheeks have a greater cardiac component in comparison with other regions of the face (nose and eyes). By using the coordinates of the *ROIe*, we excluded the eye area to reduce the artifacts produced by blinking and thus improve the SNR.

**Fig. 2:**
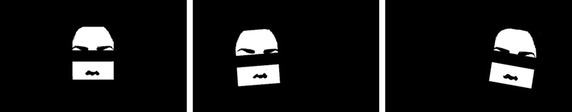
Face tracking in a subject

Huang and Dung [26] used the Viola and Jones algorithm to detect the nose in every frame of the video. This ROI detection method can be computationally expensive and not always robust if the subject makes some type of gestures. We therefore applied the Viola and Jones and KLT algorithms as a more efficient tracking method. The tracking of feature points by the KLT algorithm allowed the ROI to be detected in every frame, even if the subject performed a head tilting (Fig. 2). Thus, the ROI size was adaptable in accordance with movements of the head or changes in facial expressions. The feature points detected within the *ROIe* were eliminated to avoid changes in the ROI size caused by the blinking, which may affect the measurements.

### Video and signal processing

**Fig. 3:**
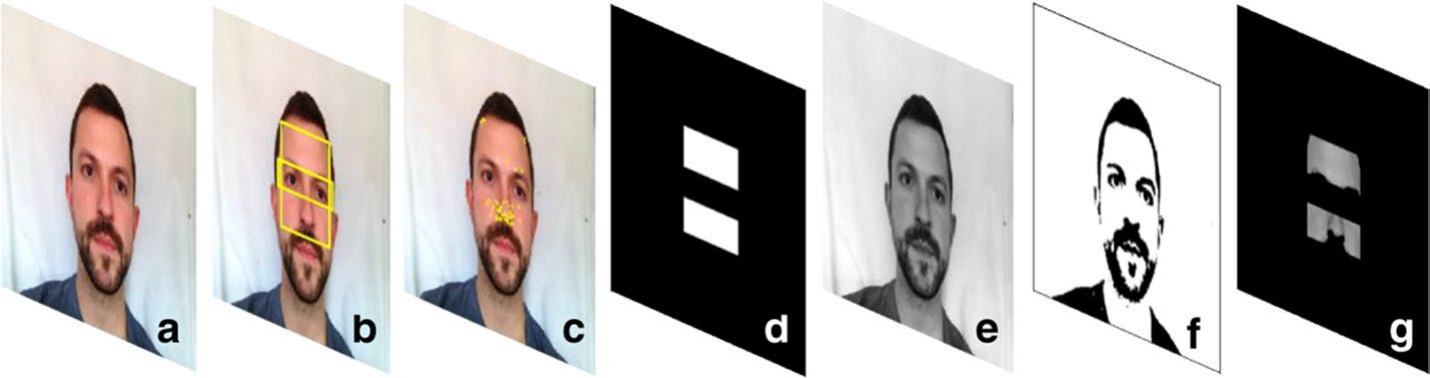
Video image processing: **a** original frame; **b** *ROIf* and *ROIe*; **c** feature points detected on frame; **d** ROI filter; **e** green channel of frame; **f** binary image; **g** multiplication resulting image from **d**–**f**. All pixels with non-zero values from **g** image were averaged across all the video sequence to obtain a raw iPPG signal. The subject gave permission to publish his photo in the paper

Video recordings and signals were processed and analysed in MATLAB^®^ (R2015b). The green channel of the video was analysed because previous studies demonstrated that it gives the best iPPG signal in comparison with the other channels [30, 31]. The objective was to analyse the pixels that correspond to the skin and the exclusion of regions that may contribute with artifacts. The frames were therefore converted into threshold-based binary images to highlight the skin from darker areas. Thus, the pixels inside the *ROIf* and corresponding to the skin were analysed, and areas such as hair, eyebrows, and beard were excluded from the analysis. The pixels outside the *ROIf* and inside the *ROIe* were also excluded. The corresponding images resulting from each frame were multiplied to obtain the final image to be processed. A representation of the video image processing is illustrated in Fig. 3.

**Fig. 4:**
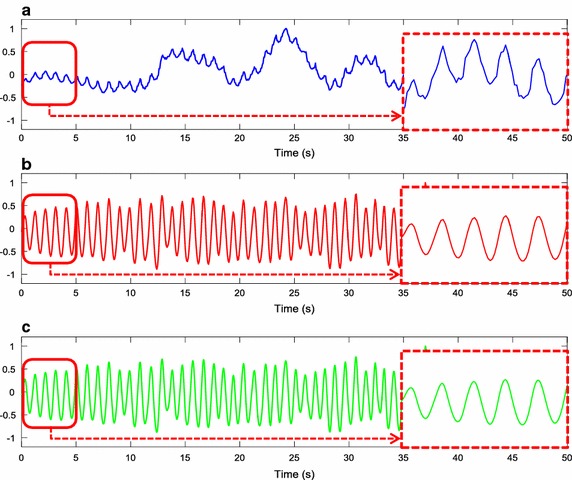
iPPG signals from a subject in motions conditions: **a** raw signal; **b** filtered signal; **c** interpolated signal

From the resulting image, all pixels with non-zero values were averaged across all the video sequence to obtain a raw iPPG signal. A first-order bandpass Butterworth filter between 0.6 and 2 Hz was applied to remove low and high frequency noise components. A cubic spline interpolation was performed to improve the temporal resolution from 15 and 60 Hz to 1 kHz. According to the recommendations by the Task Force, it is necessary to choose a minimum sampling rate in order to perform an appropriate HRV analysis [1]. An optimal range is established between 250 and 500 Hz or even higher. Therefore, the frequency of 1 kHz was chosen to record the reference signal, as well as to improve the temporal resolution of the iPPG signal. Figure 4 shows the iPPG signal results of a subject in motion conditions.

### Synchronisation of the pulse signals

**Fig. 5:**
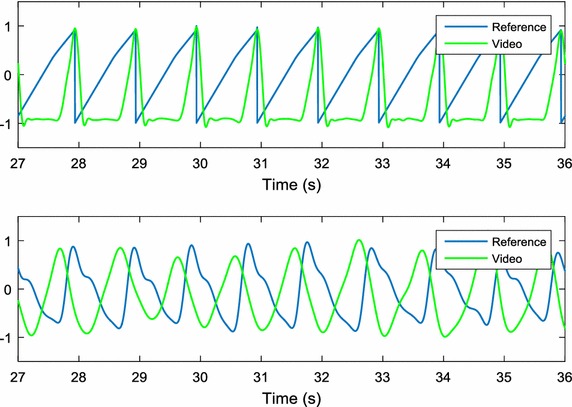
Synchronised signals: (top) sawtooth signals; (bottom) pulse signals. Once the signals were synchronised, a short delay remains between the pulse signals because the blood perfusion arrives at different times to the measuring points (face and finger)

The alignment of the pulse signals was necessary to perform a comparative PRV analysis, since the camera and the reference system were not synchronised in time. A sawtooth signal was obtained from a function waveform generator and recorded by the reference system. The sawtooth signal was also obtained by video from the green light-emitting diode (LED) connected to the function waveform generator and positioned close to the participants (Fig. 1). The LED was turned on when both systems were recording, and then turned off before recording ceased. The acquisition of the sawtooth signal by video was carried out by measuring the changes in light intensity of the LED. To achieve this, the values of the pixels where the LED was located were averaged along the sequence of video frames. This procedure results in a signal that corresponds to the sawtooth signal obtained from the function waveform generator. Due to the relationship between the acquisition method of the pulse and sawtooth signals, the time offset between both sawtooth signals is exactly the same delay as that between the pulse signals. We were therefore able to synchronise the recordings by calculating the time offset between the sawtooth signals. A representative illustration of the synchronised signals is shown in Fig. 5.

### Artifacts correction

Once the pulse signals were synchronised, the local maxima were detected in order to calculate the PP intervals. In general, the quality of the iPPG signals was excellent in both conditions, although a few subjects presented false positive and false negative measurements that affected the PP time series. Then, prior to the PRV analysis, the PP intervals falling outside established thresholds were identified as artifacts and replaced with new values. The thresholds were defined as the median ± 4 standard deviations of the PP time series. When a false positive (*false beat*) was detected, two incorrect intervals were originated in the PP time series. These erroneous measurements are usually shorter than the expected values because they correspond to one PP interval. Thus, the incorrect measurements were replaced with the sum of their corresponding values. In the case of PP measurements, those above the upper threshold, caused by a false negative (*missing beat*), were replaced with the average of the five previous PP intervals. Furthermore, the absolute values of the differences between adjacent PP measurements (PP–PP) that were above the median + 4 standard deviations of the PP–PP series were identified as outliers. These outliers were also replaced by following the same procedure as that in a false negative case.

### PRV and statistical analysis

A PRV analysis was performed according to the parameters used in a HRV analysis [1]. Some of the most common time- and frequency-domain parameters were calculated in the analysis. The time-domain components were calculated in MATLAB^®^. The Kubios HRV software (version 2.2) was used to obtain the frequency-domain parameters from the PP time series. The low-frequency (LF) and high-frequency (HF) components in normalized units (n.u.) were included in the results. These parameters were obtained by Welch’s method, which employs the FFT for the calculation of PSD.

In addition, the following statistical parameters were calculated to measure the agreement and the error between the reference system and the proposed method by video imaging:

- *Pearson’s correlation coefficient (r)* a statistical parameter used to measure the linear association between two continuous variables *x* and *y*. It is defined by:
  $$r = \frac{{\mathop \sum \nolimits_{i = 1}^{n} \left( {x_{i} - \overline{x} } \right)\left( {y_{i} - y} \right)}}{{\sqrt {\mathop \sum \nolimits_{i = 1}^{n} \left( {x_{i} - \overline{x} } \right)^{2} \mathop \sum \nolimits_{i = 1}^{n} \left( {y_{i} - \overline{y} } \right)^{2} } }}$$
  where r ranges from the − 1 to + 1 interval. A value of + 1 indicates a perfect positive association, 0 means no association and − 1 indicates a perfect negative association.
- *Intraclass correlation coefficient (ICC)* a statistical parameter that measures absolute agreement between two continuous variables:
  $$ICC = \frac{{\sigma_{T}^{2} }}{{\sigma_{T}^{2} + \sigma_{e}^{2} }}$$
  In general, ICC values below 0.4 represent poor agreement, values between 0.4 and 0.75 indicate a good agreement, and values above 0.75 represent an excellent agreement of the measurements [32].
- *Mean absolute error (MAE)* a measure that represents the average of the absolute errors between two continuous variables. It is defined as:
  $$MAE = \frac{1}{n}\mathop \sum \limits_{i = 1}^{n} \left| {p_{i} - o_{i} } \right|$$
  where *p*_*i*_ is the predicted value and *o*_*i*_ is the observed value.
- *Mean absolute percentage error (MAPE)* a measure that expresses accuracy as a percentage of the error between two continuous variables. It is defined as:
  $$MAPE = \frac{100}{n}\mathop \sum \limits_{i = 1}^{n} \left| {\frac{{p_{i} - o_{i} }}{{p_{i} }}} \right|$$
  where *p*_*i*_ is the predicted value and *o*_*i*_ is the observed value.
- *Root mean square error (RMSE)* a common measure of the differences between two continuous variables, but in comparison with MAE, RMSE it punishes large errors. It is defined as:
  $$RMSE = \sqrt {\frac{1}{n}\mathop \sum \limits_{i = 1}^{n} \left( {p_{i} - o_{i} } \right)^{2} }$$
  where *p*_*i*_ is the predicted value and *o*_*i*_ is the observed value.
- *Bland*–*Altman plot* a plot used to assess the agreement between two methods of clinical measurement [33]. Each point on the plot is represented by the mean of the measurements *x* and *y* obtained by the two methods on the X-axis, and by the difference between these measurements on the Y-axis:
  $$S\left( {X,Y} \right) = \left( {\frac{x + y}{2},x - y} \right)$$

The limits of agreement (LoA) of 95% are calculated by the mean difference ± 1.96 the standard deviation of the differences:

$$LoA \left( {95\% } \right) = \overline{d} \pm 1.96\;{\text{s}}$$

## Results and discussion

### First study: performance analysis of video cameras

**Fig. 6:**
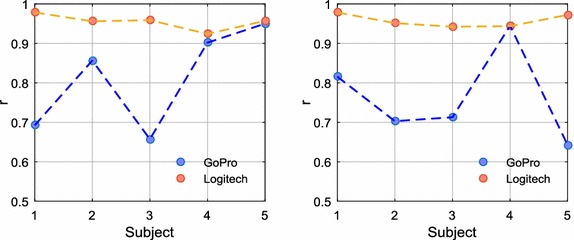
Pearson’s correlation coefficient (r) of the PP measurements obtained by the cameras: (left) stationary conditions; (right) motion conditions. All p-values < 0.001

Figure 6 shows the Pearson correlation coefficient of the PP measurements obtained by the two cameras in stationary and motion conditions. Table 2 shows the mean ± standard deviation ($\overline{x} \pm s$) results of the correlation coefficients and errors of the PP measurements. It should be pointed out that the Logitech model achieved better results than the GoPro camera in both conditions. Moreover, the results of the webcam were similar in both conditions of the study.

**Table 2:** Statistical results of the PP measurements obtained by the cameras ($\overline{x} \pm s, N = 5$)

Camera	Pearson (r)	ICC	MAE	MAPE (%)	RMSE	FP (%)	FN (%)
Stationary conditions							
GoPro	0.812 ± 0.130	0.796 ± 0.146	23.59 ± 8.02 ms	2.83 ± 0.74	35.91 ± 22.50 ms	0	0.7
			(2.11 ± 0.65 bpm)		(3.11 ± 1.18 bpm)		
Logitech	0.955 ± 0.020	0.954 ± 0.020	11.70 ± 3.92 ms	1.44 ± 0.60	16.02 ± 6.85 ms	0	0
			(1.08 ± 0.55 bpm)		(1.48 ± 0.88 bpm)		
Motion conditions							
GoPro	0.764 ± 0.119	0.737 ± 0.142	32.76 ± 8.60 ms	3.77 ± 1.16	44.31 ± 16.11 ms	0.4	1.1
			(2.59 ± 0.91 bpm)		(3.49 ± 1.58 bpm)		
Logitech	0.958 ± 0.017	0.957 ± 0.017	13.23 ± 4.52 ms	1.54 ± 0.59	16.76 ± 5.52 ms	0	0
			(1.09 ± 0.48 bpm)		(1.39 ± 0.59 bpm)		

Refer to “PRV and statistical analysis” section for parameter definitions

*FP* false positive, *FN* false negative

**Fig. 7:**
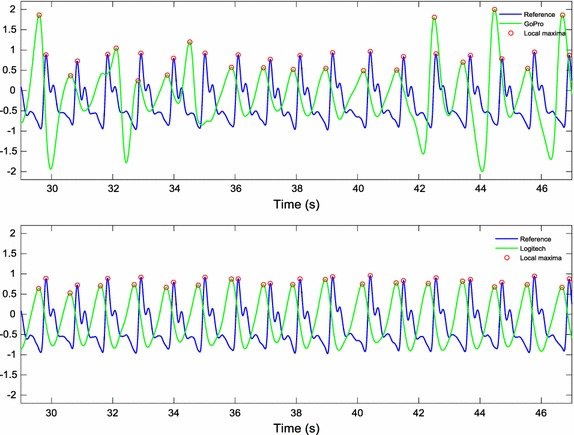
iPPG signals obtained by the cameras in simultaneous recording under lighting disturbances: (top) GoPro camera; (bottom) Logitech camera

A determining factor that greatly affected the recordings obtained by the GoPro camera was the lighting condition on a partly cloudy day. Although recordings were carried out indoors with sunlight as the only source of illumination, some unpredictable lighting changes influenced some video recordings. After video processing, it was observed that some of the signals obtained by the GoPro model were more affected by these illumination disturbances. Since we are measuring the small changes in skin colour, these disturbances cause alterations in the signal that make the acquisition of reliable measurements difficult. A comparative illustration of the iPPG signals obtained by the two cameras under lighting disturbances is shown in Fig. 7.

Although the lighting condition is an external factor, it is very important to take into account because it may affect the camera sensors differently. Lighting conditions should also be taken into consideration if the application in real environments is contemplated. Additionally, a slight blur in the videos obtained by the GoPro model was observed that could affect the measurements. This issue arose because the face was positioned at a short distance from the camera and the impossibility to adjust the focus. The Logitech model did not present this problem because the focus was manually adjusted before the recordings.

Despite the difference in frame rate of the cameras, it appears that this parameter has little effect on the measurements. As shown, the signals acquired with the higher frame rate did not yield better results, at least in stationary conditions and without the presence of lighting disturbances. Thus, the better overall results obtained by the webcam and the possibility of making manual settings were decisive for choosing this camera in the later recordings.

### Second study: PRV analysis

#### Stationary conditions analysis

**Fig. 8:**
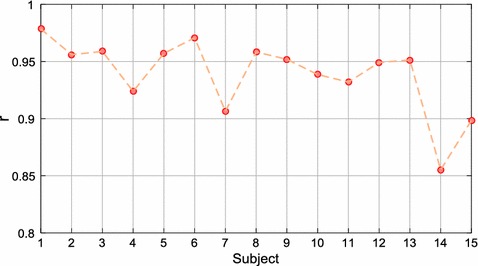
Pearson’s correlation coefficient (r) of the PP measurements of each subject in stationary conditions. All p-values < 0.001

Once the first part of the study was completed, the second part was conducted in the same conditions using the chosen camera. Figure 8 shows the Pearson correlation coefficient of the PP measurements of each subject. Most of the subjects obtained correlation coefficients above 0.9. Table 3 shows the $\overline{x} \pm s$ of the correlation coefficients and errors of all subjects. The Pearson’s and ICC measures were 0.939 ± 0.032 and 0.937 ± 0.033, respectively.

**Table 3:** Statistical results of the PP measurements in stationary conditions ($\overline{x} \pm s, N = 15)$

Pearson (r)	ICC	MAE	MAPE (%)	RMSE	FP (%)	FN (%)
0.939 ± 0.032	0.937 ± 0.033	15.04 ± 4.30 ms	1.78 ± 0.60	19.45 ± 5.52 ms	0	0.1
		(1.30 ± 0.56 bpm)		(1.70 ± 0.75 bpm)		

Refer to “PRV and statistical analysis” section for parameter definitions

*FP* false positive, *FN* false negative

Some participants were observed to have achieved better results than others (Fig. 8). Since the analysis was carried out in stationary conditions, some factors such as the different anatomical and physiological characteristics of the participants may have influenced the measurements. Kumar et al. [24] performed PRV estimations for different skin tones (fair, olive and brown) in which 4 subjects were analysed according to skin category. The fair and olive skin tones presented quite similar results with RMSE values of 13.61 and 13.36 ms, respectively, while for the brown category a higher RMSE of 48.91 ms was obtained. Based on the results obtained from the fair and olive skin categories and, the similarity of skin tone of the subjects in our study, the differences in the results between subjects may not be due to this physical characteristic. There are other factors besides skin colour which may also influence the measurements, such as blood pressure, heart rate, respiration, etc. Moreover, although we asked the participants to keep still during the recording, some had difficulties in remaining motionless or avoiding facial expressions. Sunlight affected some subjects causing a major blinking in them.

With the aim of conducting the analysis in as fair a way as possible, the results of this study are contrasted with data reported in reference works carried out in similar conditions. Some authors have reported statistical results of the PP measurements (or IBIs) obtained in stationary conditions [20, 23–25], whose corresponding results are summarized in Table 1. By examining these results and the corresponding data presented in Table 3, it is noted that our method obtained good results. The PP time series measured by our method achieved a higher correlation in comparison with the result presented by Bousefsaf et al. With regard to the error results reported by the authors, only the study of Kumar et al. achieved a lower error compared with the 19.45 ms obtained by our method. It is important to note that our study presented a larger sample size than the cited works. Thus, the sample size should also be taken into account since the results may vary considerably between subjects, even if there is a minimal presence or and absence of motion artifacts (Fig. 8).

**Fig. 9:**
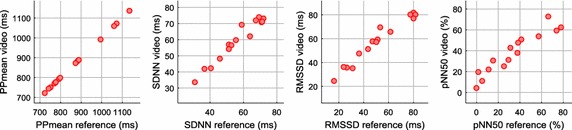
Correlation scatter plots of the time-domain parameters of PRV in stationary conditions: **a** PPmean; **b** SDNN; **c** RMSSD; **d** pNN50. Refer to Table 4 for r and ICC results

Figure 9 shows the correlation scatter plots of the PRV time-domain parameters and Table 4 presents the results of HR and PRV parameters obtained in stationary conditions by several authors and our method. High correlation values were obtained by our method, most of which achieved results above 0.9. HR was estimated by calculating the mean of the PP measurements in beats/min (bpm). In contrast with the results presented by Poh et al. [17], the RMSE of HR was reduced from 1.24 to 0.07 bpm and the frequency-domain parameters also presented lower errors.

**Table 4:** Statistical results of HR and PRV parameters in stationary conditions

Study	Statistical parameter	Condition/method	Parameter							
			HR (bpm)	PPmean (ms)	SDNN (ms)	RMSSD (ms)	pNN50 (%)	LF (n.u.)	HF (n.u.)	LF/HF
Poh et al. [17]	r		1.00*	–	–	–	–	0.92*	0.92*	0.88*
	RMSE		1.24	–	–	–	–	12.3	12.3	1.1
Sun et al. [18]	r		–	0.998*	0.874*	–	–	0.971*	0.978*	0.875*
McDuff et al. [19]	r	(1)	1.00^x^	–	–	–	–	0.87^x^	0.87^x^	0.86^x^
		(2)	1.00^x^	–	–	–	–	0.97^x^	0.97^x^	0.95^x^
Moreno et al. [21]	ρ_c_	(2)	–	0.9999	0.9108	0.5180	0.5385	0.7934	0.7838	0.3186
Alghoul et al. [22]	r	(1)	–	0.999*	–	–	–	0.8*	0.84*	0.74*
		(2)	–	0.999^x^	–	–	–	0.831^x^	0.789^x^	0.256^x^
	MAE	(1)	0.006	0.005	0.08	–	–	0.34	0.57	0.419
		(2)	0.006	0.006	0.1	–	–	0.28	0.76	1.69
Bousefsaf et al. [23]	r	(1)	1.00*	–	–	–	–	–	–	–
Huang and Dung [26]	MAE	(1)	–	–	2.01	4.33	–	–	–	–
Our method	r		1.0000*	1.0000*	0.9750*	0.9791*	0.9350*	0.9824*	0.9824*	0.9521*
	ICC		1.0000*	1.0000*	0.9491*	0.9208*	0.8996*	0.9546*	0.9546*	0.7593*
	MAE		0.06	0.51	3.49	7.13	8.65	4.78	4.77	0.31
	RMSE		0.07	0.69	4.26	8.29	10.35	6.20	6.20	0.61

* p < 0.001; ^x^Unspecified *p* value; – parameter was not reported; ρ_c_: concordance correlation coefficient. The MAE results from [26] are calculated in this work to obtain a mean value of the individual results. Refer to “PRV and statistical analysis” section for parameter definitions. Refer to Table 1 for the conditions/method of the study. (Note: the results reported in [22] appear to make no sense, likely, due to a transcription error. Even so, the results are included in this table)

Sun et al. [18] acquired the recordings by focusing on the palm of the subject’s hand, with a resolution of 384 × 256 pixels at 200 fps. The PRV parameters achieved very good correlation values, although the correspondence of the SDNN and LF/HF components was considerably lower in comparison with our results. Despite the higher frame rate, the results did not prove to be better. The lower video resolution and the analysed ROI may have affected the measurements. Also, it is important to note that the sample size was larger in our work, but the recording length was longer in the study of Sun et al.

In the work of McDuff et al. [19], besides the HR measurement, they presented results of the frequency-domain parameters of PRV at rest and under stress. They used a five band digital camera in which the combination of cyan, green, and orange (CGO) bands yielded a higher correlation with the reference sensor. Curiously, the results under stress achieved higher correlation values than those obtained at rest. The best results obtained of this work were similar in comparison with the data achieved in our study, in which only the green channel of the video was analysed.

Moreno et al. [21] reported some of the most common time and frequency domain parameters; it is clear that the RMSSD, pNN50, and frequency-domain parameters yielded lower correlation results in comparison with our method. The ICC measure is almost identical to the concordance correlation coefficient (ρ_c_) [34] reported by Moreno et al. and, in general, ICC values above 0.75 represent an excellent reliability in the measurements [32]. One of the factors that may have an influence on the measurements is the video resolution. Moreno et al. recorded the videos with a resolution of 640 × 480 pixels, the same as Poh et al., which is lower in comparison with the video resolution of our work. Moreover, as shown in the performance analysis of cameras, it seems that the higher frame rate of 30 fps has not been significant in improving the measurements.

The study of Alghoul et al. [22] presented a comparison between two approaches to measure HRV parameters from the face in stationary conditions. On one side, the results reported in the study showed that ICA-based method yielded lower errors in the HF and LF/HF components and, on the other hand, the LF parameter achieved better results with the EVM-based approach. Although the correlation values presented by Alghoul et al. were lower than those achieved by our method, some corresponding errors were better than the data in our study, which appear to make no sense, likely, due to a transcription error. Even so, the results are included in this work in Table 4.

Huang and Dung [26] only presented single results of HRV parameters using absolute error measures. Therefore, the MAE results shown in our study were calculated to obtain a mean value of the individual results. These data are slightly better than the corresponding results achieved by our method, but using a smaller sample size in comparison with our analysis. Thus, the application of the proposed method by Huang and Dung appears promising in the acquisition of measurements in stationary conditions. It would be interesting the application of this approach to estimate time and frequency domain parameters of HRV with a larger sample size.

#### Motion conditions analysis

Although HRV is normally measured at rest, recently, the interest of measuring physiological variables in everyday activities by using alternative methods has increased. Most of the daily life scenarios present the motion inherently, which must be considered because it may affect the measurements, especially when is used the video imaging technique. For example, the drowsiness in drivers is one the causes of traffic accidents all over the world. Therefore, some works [35, 36] have proposed the measurement of HRV parameters in drivers during alert and drowsy or fatigued periods, in which some parameters showed significant differences between both states. Other studies have proposed the analysis by video to measure physiological parameters while driving [37–39] and other ones to detect cardiac arrhythmias [40, 41]. Thus, the video imaging technique may eventually become a method to detect these events in drivers with the aim of preventing traffic road accidents. Also, since the HRV analysis is an excellent indicator of physical and psychological stress, this technique may be used to evaluate the stress level of people during working hours or as a complement in a polysomnography study.

**Fig. 10:**
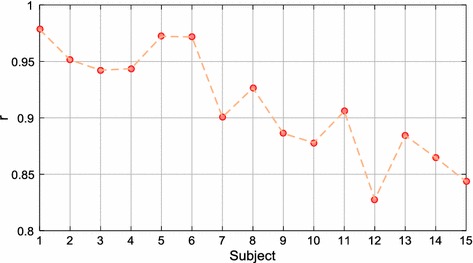
Pearson’s correlation coefficient (r) of the PP measurements of each subject in motion conditions. All p-values < 0.001

In this part of the study, the same analysis was conducted as that for stationary conditions. Figure 10 shows the Pearson correlation coefficient of the PP measurements of each subject. In general, the correlation results were lower in comparison with the stationary conditions, but they also varied according to the participant; even so, results of above 0.9 were obtained with several subjects. Table 5 shows the $\overline{x} \pm s$ of the correlation coefficients and errors. The Pearson and the ICC measures were 0.912 ± 0.048 and 0.911 ± 0.050, respectively.

**Table 5:** Statistical results of the PP measurements in motion conditions ($\overline{x} \pm s, N = 15)$

Pearson (r)	ICC	MAE	MAPE (%)	RMSE	FP (%)	FN (%)
0.912 ± 0.048	0.911 ± 0.050	17.11 ± 5.33 ms	1.98 ± 0.61	21.56 ± 6.41 ms	0	0
		(1.41 ± 0.49 bpm)		(1.79 ± 0.63 bpm)		

Refer to “PRV and statistical analysis” section for parameter definitions

*FP* false positive, *FN* false negative

In comparison with the stationary posture, it is noted how some subjects obtained similar results; on the other hand, some subjects did not present results as good as those under the first condition. The motion conditions of our study consisted of lateral and forward/backward movements that added the difficulty of obtaining the iPPG signal at different positions and distances from the camera. These are common movements that are performed while working in front of a PC or in other activities with similar moves. Moreover, these movements presented the particularity that they were performed at a steady and non-stop pace. The employed tracking algorithm performed very well in accordance with the different movements carried out by the subjects. However, despite we asked the participants to execute the same type of movements, in practice their gestures tended to vary slightly; the type of head inclination, the pace of the movements or the distance between the camera and the face during the forward/backward motions are some examples of these variations. Thus, it should be pointed out that some particular movements may significantly affect the iPPG signals, thereby making the acquisition of PP measurements more difficult.

To date, most of the works of the state of the art have obtained HRV measurements in stationary conditions, therefore, the number of studies that have performed an in-depth statistical analysis in motion conditions is very limited. In the literature, there are works that have measured physiological parameters by video in motion scenarios [42–46], but with assessments that make difficult a comparative analysis with the studies reviewed in this paper. In other works, some authors have reported statistical results of the IBIs obtained in motion conditions [23–25], whose corresponding results are summarized in Table 1.

Bousefsaf et al. [23] obtained IBIs measurements with predefined head movement conditions. Although the results obtained in a sitting still and calm condition were better, it seems that the motion scenario did not affect to a great extent the measurements. These results obtained a lower correlation than that presented in Table 5. The motion-tolerant method proposed by Bousefsaf et al. appears to perform well in both conditions, although the low video resolution of the recordings could have influenced the measurements.

The study of Kumar et al. [24] performed the acquisition of signals in three natural motion scenarios. The reading scenario achieved an RMSE of 55.34 ms and the watching video and talking activities obtained the higher errors. Although none of the three motion scenarios is equivalent to the motion conditions of our study, the reading scenario may have a closer resemblance due to a greater presence of moderated movements. This may explain the better result achieved by this scenario. Nevertheless, a reasonably higher error was obtained in comparison with the result achieved in our study (21.56 ms). Besides the difference of the motion scenarios, it is also important to take into consideration the difference in sample size of both studies, as mentioned in other cases.

Antink et al. [25] performed a beat-to-beat estimation by means of different signals and their fusion. Within these signals, we have focused on the video signal obtained from the changes of the skin colour. The conditions of the third trial of the study consisted in the acquisition of measurements during reading without further instructions, unlike the second trial (reading-task without motion). Thus, the difference in results between the trials 2 and 3 is, likely, because of the presence of motion artifacts during the reading-task. The MAE results shown in Table 1 are calculated in this work to obtain a mean value of the individual results reported by Antink et al. in each trial. The MAE achieved by our method in motion conditions was lower than the mean error calculated in the third trial, as well as in comparison with the recordings performed without motion.

**Fig. 11:**
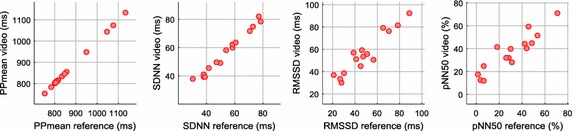
Correlation scatter plots of the time-domain parameters of PRV in motion conditions: **a** PPmean; **b** SDNN; **c** RMSSD; **d** pNN50. Refer to Table 6 for r and ICC results

Likewise, the acquisition of HR and PRV parameters was carried out for purposes of comparison with data reported in other works and the results obtained in stationary conditions. Figure 11 shows the correlation scatter plots of the time-domain parameters of PRV and Table 6 presents the results of HR and PRV parameters obtained in motion conditions by other authors and our method. In our study, most of the parameters obtained correlation results above 0.9, but with lower values in comparison with the stationary conditions. The RMSSD, pNN50, and frequency-domain parameters yielded the lower correlation results, in particular, the LF/HF ratio.

**Table 6:** Statistical results of HR and PRV parameters in motion conditions

Study	Statistical parameter	Condition method	Parameter							
			HR (bpm)	PPmean (ms)	SDNN (ms)	RMSSD (ms)	pNN50 (%)	LF (n.u.)	HF (n.u.)	LF/HF
Bousefsaf et al. [23]	r	(2)	1.00*	–	–	–	–	–	–	–
Huang and Dung [26]	MAE	(3)	–	–	11.94	24.00	–	–	–	–
		(4)	–	–	6.05	12.37	–	–	–	–
Our method	r		1.0000*	1.0000*	0.9896*	0.9463*	0.9204*	0.9288*	0.9302*	0.8548*
	ICC		1.0000*	1.0000*	0.9766*	0.8898*	0.8560*	0.9247*	0.9265*	0.4468**
	MAE		0.06	0.65	2.65	7.81	7.80	5.54	5.47	1.10
	RMSE		0.08	0.89	3.34	9.34	10.30	7.44	7.34	3.28

* p < 0.001; ** p < 0.05; – parameter was not reported. The MAE results from [26] are calculated in this work to obtain a mean value of the individual results (note: the recordings of the occasional and frequent motion categories [26] were performed by the same subject). Refer to “PRV and statistical analysis” section for parameter definitions. Refer to Table 1 for the conditions of the study

Huang and Dung [26] recently presented a study with measures of HRV parameters obtained during occasional and frequent motion. Only single results of HRV parameters using absolute error measures were reported in their study. The sample size of the motion categories were N = 3 and N = 2, respectively, which were performed by the same subject. In comparison with both categories, the MAE results of our study were lower than the data calculated from the results reported by them.

The occasional motion category consisted of three different recordings. One of them presented motions that were very similar to some of the movements performed in our study, in which the subject moved away from the camera and then moved back. In another recording, the subject shook the head three times but it was not specified how these movements were performed and, in the last one the subject talked and turned the head. In the case of the frequent motion category, the two recordings presented movements that were repeated along the video sequence.

The occasional motion category presented a higher MAE than the corresponding data of the frequent motion condition. This occurred because of the third recording of the occasional motion category obtained a considerable absolute error of 28.87 ms, likely, due to the presence of motion artifacts caused by the talking scenario. The face detection method could also affect the measurements because it is not always robust if the subject makes some type of gestures. Moreover, the small sample size with one subject and the motion conditions of the recordings, which were completely different from each other, make the data not suitable for obtaining reliable statistical results.

#### Bland–Altman plot analysis

**Fig. 12:**
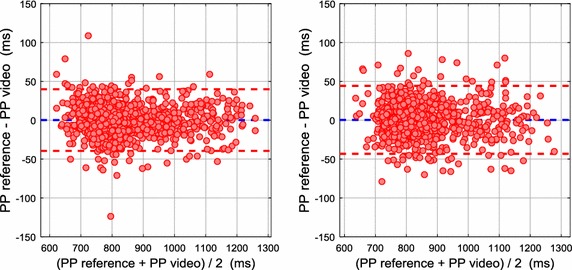
Bland–Altman plots with limits of agreement (LoA) of 95% representing the agreement between the PP measurements obtained by the reference system and the video (data of all subjects combined in one dataset): left: stationary conditions with LoA from − 39.47 to 39.90 ms (− 3.81 to 3.69 bpm); right: motion conditions with LoA from − 43.28 to 44.18 ms (− 3.84 to 3.72 bpm)

Figure 12 shows the Bland–Altman plots representing the agreement between the PP measurements obtained by the reference system and the video in stationary and motion conditions. The stationary posture achieved LoA from − 39.47 to 39.90 ms (− 3.81 to 3.69 bpm), and the motion scenario obtained LoA from − 43.28 to 44.18 ms (− 3.84 to 3.72 bpm). No large differences were found between both conditions of the study, and also, no systematic errors were identified in the measurements. According to the Bland–Altman analysis, if the differences obtained by the measurement systems are not regarded as clinically important, both may be used interchangeably.

Within the studies cited in previous sections, some of them obtained Bland–Altman plots of HR measurements in stationary and motion scenarios. The best results obtained in these works are summarized in Table 7.

**Table 7:** Bland-Altman results of HR in stationary and motion conditions

References	Signal analysis	Stationary conditions			Motion conditions		
		Mean bias (bpm)	Lower LoA (bpm)	Upper LoA (bpm)	Mean bias (bpm)	Lower LoA (bpm)	Upper LoA (bpm)
Poh et al. [13]	FD	− 0.05	− 4.55	4.44	0.64	− 8.35	9.64
Monkaresi et al. [16]	FD	0.86	− 1.99	3.71	0.06	− 7.09	7.21
Bousefsaf et al. [23]	TD	− 0.03	− 4.22	4.16	0.02	− 4.96	4.99
Kumar et al. [24]	FD	− 0.02	− 0.75	0.72	0.17	− 4.52	4.86
Our method	TD	− 0.06	− 3.81	3.69	− 0.06	− 3.84	3.72

*LoA* limits of agreement of 95%, *FD* frequency-domain, *TD* time-domain

Poh et al. obtained the lowest agreement in both conditions among the analysed studies. In the work by Monkaresi et al. the ICA & kNN method achieved the best accuracy in motion conditions, with LoA from − 7.09 to 7.21 bpm. The LoA obtained by Bousefsaf et al. were slightly wider than those of our study in both conditions, where these works obtained the HR measurements by a time-domain signal analysis. Kumar et al. obtained LoA from − 5.73 to 6.70 bpm for the three motion scenarios (reading + watching + talking), and from − 4.52 to 4.86 bpm for the non-talking scenario (reading + watching). The study by Kumar et al. achieved the best accuracy when compared with the other works, especially in stationary conditions, although our method achieved a slightly better result in motion. It is important to take into account that the measurements were acquired by different signal analysis and motion scenarios.

#### Video PRV analysis for detecting physical and psychological disorders

As initially mentioned, HRV is a parameter that is related to physical and psychological disorders. Thayer et al. [5] reviewed several studies that have analysed the association between HRV and cardiovascular disease risk factors, such as diabetes, cholesterol, smoking, physical inactivity, obesity, and work stress. In this work, the authors provided strong evidence to support that a low HRV is closely related to the major risk factors that lead to the development of cardiovascular diseases. By analysing these studies, it was noted the difficulty in establishing a clear distinction between subjects who belong to different examined groups, especially when there are small differences. Although the HRV parameters presented significant differences between groups, the dispersion of the data may cause an overlap leading to a disorder detection error.

Even so, we may assess the effectiveness of our method for detecting physical or psychological disorders by considering only the mean values of HRV parameters obtained by some studies. Rossy and Thayer [47] analysed the ANS control of the heart in low-fit and high-fit people by examining the HRV parameters. Some HRV measurements obtained by these groups were: RRmean (low-fit = 781 ms, high-fit = 899 ms) and SDNN (low-fit = 65 ms, high-fit = 85 ms). If we consider the MAE results achieved in these parameters by our work, the HRV measurements obtained by the groups would be distinguishable by the proposed method.

In another study, Singh et al. [48] examined the association of HRV with blood glucose levels in a large community-based population. The results showed that the normal fasting glucose group obtained an ln LF/HF ratio of 1.22, unlike the diabetes mellitus group that obtained a value of 1.08. Due to the little difference between the mean results of the groups, and considering the error achieved in our study, in this case, the method would not be able to distinguish between LF/HF values obtained by the subjects of the study. Therefore, the effectiveness for detecting disorders will depend not only on the degree of dispersion of the data obtained by the examined groups, but also on the measurement error of the method.

## Conclusions

In this work, a video PRV analysis was carried out in stationary and motion conditions. The analysis performed provides a better insight of the scope and limitations of the proposed method in comparison with other related studies. An initial performance analysis of cameras validates the use of the webcam as a better option in this study. It is shown that it is possible to obtain good results by using a video recorded at a lower frame rate. Furthermore, the higher the frame rate, the higher the computational cost of the video processing. In addition to analysed factors such as frame rate and motion, it is also shown that the measurements could be affected by the physiological characteristics of the participants, lighting conditions, focus, resolution or the measuring distance.

The statistical analysis shows a good agreement between the reference system and the proposed method. In stationary conditions, the results of PRV parameters are improved by our method in comparison with data reported in related works. Most of the PRV parameters also achieve good correlation results in the motion analysis, but with lower values in relation to the stationary conditions. An overall comparative analysis of PRV parameters in motion conditions was more limited due to the lack of studies or studies containing insufficient data analysis. The larger sample size and the in-depth statistical analysis of our study provide greater reliability of the data. Based on these results, the method proposed herein could provide a low-cost, contactless and reliable alternative for measuring HR or PRV parameters in non-clinical environments. Furthermore, it is shown that this non-invasive technique may eventually become an instrument to detect physical or psychological disorders in the future. Clearly, its use for this purpose will depend on the progress of the technique over the coming years. However, the need of more medical tests will always be required to make an accurate diagnosis.

This study is a first assessment of the proposed method in motion conditions, with movements that can be performed while working in front of a PC or in other activities with similar moves. In general, the results between stationary and motion conditions do not differ significantly, although it is important to note that the measurements were carried out following specific movements. It is therefore necessary to conduct an evaluation of the proposed method considering a wider variety of motion (movements in the 6 degrees of freedom with more than 1 degree of freedom simultaneously), as well as the development of more robust algorithms in the presence of larger movements (more than 5 pixel/frame). However, with the current results, it could be applied to different scenarios where the amount of movement is reduced, as for example in driving, working at the office or during sleep. Consequently, the acquisition of measurements in more real scenarios, longer recordings, and the possibility of a real-time implementation constitute some of the objectives for future work.

## Abbreviations

HRV: heart rate variability
ANS: autonomic nervous system
RR: time interval between consecutive heartbeats
HR: heart rate
iPPG: imaging photoplethysmography
PRV: pulse rate variability
CCD: charge-coupled device
ROI: region of interest
ICA: independent component analysis
FFT: Fast Fourier Transform
HCI: human–computer interaction
kNN: k-nearest neighbor
IBIs: interbeat intervals
PSD: power spectral density
PP: pulse-to-pulse time interval
SNR: signal-to-noise ratio
C-rPPG: chrominance-based remote PPG
CWT: continuous wavelet transform
EVM: Eulerian Video Magnification
FD: frequency-domain
TD: time-domain
fps: frames per second
LED: light emitting diode
PP-PP: difference between adjacent PP measurements
LF: low-frequency
HF: high-frequency
n.u.: normalized units
r: Pearson’s correlation coefficient
ICC: intraclass correlation coefficient
MAE: mean absolute error
MAPE: mean absolute percentage error
RMSE: root mean square error
LoA: limits of agreement
bpm: beats/min
ρ_c_: concordance correlation coefficient
FP: false positive
FN: false negative
